# Minimally invasive surgical approach versus open procedure for pancreaticoduodenectomy: A systematic review and meta-analysis

**DOI:** 10.1097/MD.0000000000008619

**Published:** 2017-12-15

**Authors:** Shunda Wang, Ning Shi, Lei You, Menghua Dai, Yupei Zhao

**Affiliations:** Department of General Surgery, Peking Union Medical College Hospital, Chinese Academy of Medical Sciences and Peking Union Medical College, Beijing, People's Republic of China.

**Keywords:** meta-analysis, minimally invasive pancreaticoduodenectomy, open pancreaticoduodenectomy

## Abstract

**Background:**

Minimally invasive pancreaticoduodenectomy (MIPD) remains one of the most challenging abdominal procedures. Safety and feasibility remain controversial when comparing MIPD with open pancreaticoduodenectomy (OPD). The aim of this systematic review and meta-analysis was to evaluate the feasibility and safety of MIPD versus OPD.

**Methods:**

A systematic review of the literature was performed to identify studies comparing MIPD and OPD. Postoperative complications, intraoperative outcomes and oncologic data, and postoperative recovery were compared.

**Results:**

There were 27 studies that matched the selection criteria. Totally 1306 cases of MIPD and 5603 cases of OPD were included. MIPD was associated with a reduction in postoperative hemorrhage (odds ratio [OR] 1.60; 95% confidence interval [CI] 1.03–2.49; *P* = .04) and wound infection (OR 0.44, 95% CI 0.30–0.66, *P* < .0001). MIPD was also associated with less estimated blood loss (mean difference [MD] −300.14 mL, 95% CI −400.11 to −200.17 mL, *P* < .00001), a lower transfusion rate (OR 0.46, 95% CI 0.35–0.61; *P* < .00001) and a shorter length of hospital stay (MD −2.95 d, 95% CI −3.91 to −2.00 d, *P* < .00001) than OPD. Meanwhile, the MIPD group had a higher R0 resection rate (OR 1.45, 95% CI 1.18–1.78, *P* = .0003) and more lymph nodes harvested (MD 1.34, 95% CI 0.14–2.53, *P* = .03). However, the minimally invasive approach proved to have much longer operative time (MD 71.00 minutes; 95% CI 27.01–115.00 minutes; *P* = .002) than OPD. Finally, there were no significant differences between the 2 procedures in postoperative pancreatic fistula (*P* = .30), delayed gastric emptying (*P* = .07), bile leakage (*P* = .98), mortality (*P* = .88), tumor size (*P* = .15), vascular resection (*P* = .68), or reoperation rate (*P* = .11).

**Conclusions:**

Our results suggest that MIPD is currently safe, feasible, and worthwhile. Future large-volume, well-designed randomized controlled trials (RCT) with extensive follow-up are awaited to further clarify this role.

## 1 Introduction

During the past decade, laparoscopic surgery has played a prominent role in the general surgical field. Currently, it may be true that most surgical procedures could be performed using the laparoscopic approach. This approach also has unique advantages in selected patients in terms of shortened hospital stay, fewer postoperative morbidities, and enhanced recovery.^[1,2]^ For pancreatic surgery, pancreaticoduodenectomy (PD) is now considered to be the most widely employed surgical procedure for the treatment of pancreatic head and periampullary tumors.^[3]^ PD is recognized as challenging for surgeons due to the complex intra-abdominal dissection and reconstruction of the alimentary tract, as well as risky for patients due to consistent perioperative morbidity and mortality. In 1935, Whipple was the first to finish the pancreaticoduodenectomy for the patient with carcinoma of the ampulla of Vater,^[4]^ which has since been widely used with continuous development. In 1994, Gagner and Pomp^[5]^ published the first article introducing the laparoscopic PD. Although PD requires complex techniques and a long learning curve, it probably brought less trauma for patients compared with open PD (OPD). Nearly 10 years later, Giulianotti performed the first PD in a robotic manner in 2003.^[6]^ The robotic surgical system, a recently emerging technology, covers the intrinsic shortages of laparoscopy, including lack of tactile sensation, instrument crowding, 2-dimensional imaging, and restricted instrument movement inside the abdominal cavity. Here, we combined laparoscopic PD and robotic PD as minimally invasive pancreaticoduodenectomy (MIPD). Many famous pancreatic institutions around the world have already conducted MIPD, and expert surgeons have evaluated the minimally invasive PD and open PD in terms of safety and efficiency.^[7–10]^ However, these reports have all been based on single-institution experiences with a lack of randomized controlled trials (RCT). Compared with other published meta-analyses, our study included all of the literature on this issue which had been published to date, and included commonly used English databases and Chinese databases. Our study is the most comprehensive. In addition, former meta-analyses showed different conclusions in certain outcomes. Therefore, given the large amount of published evidence and conflicting results, the aim of this study was to systematically review and meta-analyze the studies that have compared MIPD with OPD, which could provide high-quality data for clinical practice.

## 2 Method

### 2.1 Search strategy

We performed our systematic review and meta-analysis in accordance with Preferred Reporting Items for Systematic Reviews and Meta-Analyses (PRISMA) guidelines.^[11]^ A comprehensive literature search was performed in electronic databases including Medline, PubMed, Embase, the ISI Web of Knowledge, the Cochrane Collaboration Central Register of Controlled Clinical Trials, Cochrane Systematic Reviews, CNKI, and Wan Fang (Chinese full-text database) for reports published prior to December 2016 without language restrictions. The following search terms were included but were not limited to: “pancreaticoduodenectomy,” “PD,” “Whipple procedure,” “laparoscopic/laparoscopy,” “robotic,” “Da Vinci,” and “minimally invasive.” References cited in the selected articles were also assessed to identify relevant studies in case studies that were missed during the initial database searches. The “related articles” function was used to broaden the search, and all abstracts, studies, and citations scanned were reviewed. If needed, investigators and experts in the field of pancreatic surgery were contacted to ensure that all relevant studies were identified. All analyses were based on previous published studies; thus, no ethical approval and patient consent are required.

### 2.2 Inclusion and exclusion criteria

Studies (cohort or case-control) included in our analysis were required to: compare characteristics and perioperative outcomes of patients undergoing MIPD and open pancreaticoduodenectomy (OPD). When authors and/or institutions overlapped between 2 or more studies, only the most recent study was considered. The exclusion criteria were noncomparable studies, nonhuman studies, experimental trials, review articles, editorials, letters, and case reports.

### 2.3 Outcomes of interest

All studies were abstracted for the following essential data: patient baseline characteristics, tumor characteristics, types of procedure (laparoscopic pancreaticoduodenectomy, robotic pancreaticoduodenectomy, OPD), intraoperative outcomes (operative time and intraoperative blood loss), postoperative complications (pancreatic fistula, delayed gastric emptying [DGE], wound infection, hemorrhage, and bile leakage), extension of lymphadenectomy, R0 resection, postoperative recovery time (length of hospital stay), reoperation rate, and postoperative mortality.

### 2.4 Data extraction and quality assessment

Two authors independently screened the title and abstract of each publication for potentially eligible studies. Then, full articles of eligible trials were obtained for detailed evaluation. Each study was independently assessed by 2 reviewers for inclusion or exclusion. Data regarding the following variables were extracted from the selected studies: first author, publication year, study country, study design, number of patients, characteristics of the study population, surgical procedures, postoperative management, and intraoperative and postoperative outcomes. The accuracy of the extracted data was further adjudicated by a third author. Disagreements on study selection and data extraction were resolved by team discussion.

### 2.5 Statistical analysis

This meta-analysis was performed in accordance with recommendations from the Cochrane Collaboration and Meta-analysis of Observational Studies in Epidemiology (MOOSE) guidelines.^[12,13]^ For baseline characteristics, we applied the chi-squared test for categorical variables and Student *t* test for continuous variables. The mean and standard deviation (SD) of the continuous variables were estimated using the median, range, and number of patients.^[14]^*P* values <.05 were considered statistically significant. Dichotomous variables were analyzed using odds ratio (OR). Each study was weighted by means of sample size and was reported with 95% confidence intervals (CI). With respect to the outcomes, data from the original articles were extracted and analyzed using Review Manager 5.1 software (Cochrane Collaboration). The *I*^2^ index was used as an indicator of between-study heterogeneity. *I*^2^ values ranged from 0% to 100% (*I*^2^ = 0–25%, no heterogeneity; *I*^2^ = 25–50%, moderate heterogeneity; *I*^2^ = 50–75%, high heterogeneity; *I*^2^ = 75–100%, extreme heterogeneity).^[15,16]^ In this case, the fixed-effects model was adopted mostly^[17]^; otherwise, the random-effects model was used.^[18]^ Publication bias was assessed using funnel plots, and the tests developed by Egger and Begg, which calculate funnel plot asymmetry on the natural logarithm scale of the OR based on linear regression, were used to assess asymmetry of the funnel plots. Statistical significance was considered when the *P* value was <.05.^[19,20]^

## 3 Results

### 3.1 Description of included trials in the meta-analysis

**Figure 1:**
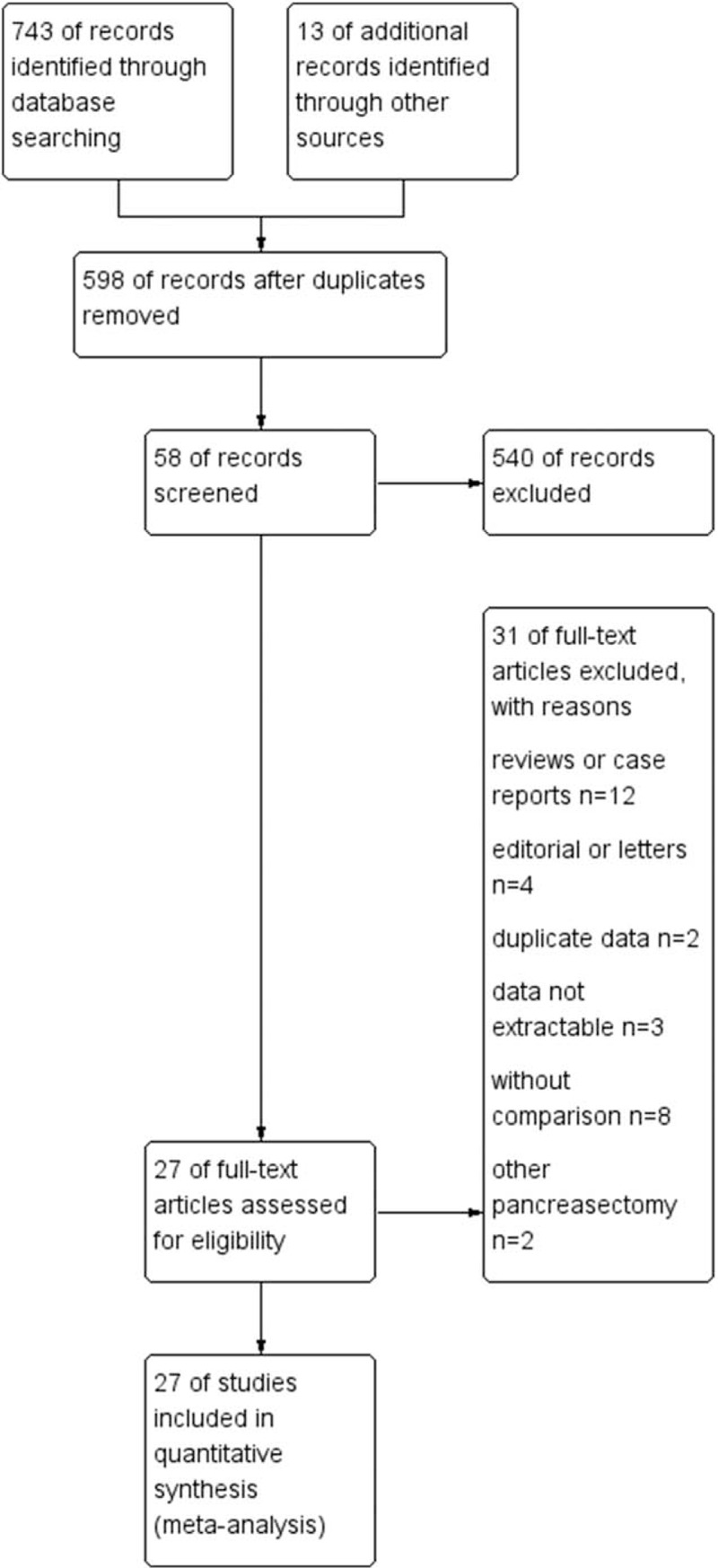
A PRISMA flow diagram depicting the selection process.

The searches in PubMed, Embase, Medline, the Cochrane Library and Chinese databases identified 756 abstracts published before December 2016. After excluding duplicates, 598 articles remained. After screening titles and abstracts, 540 articles were excluded for irrelevancy. Of the remaining 58 articles, 12 articles were reviews or case reports, 4 were editorials or letters, 2 had duplicate data, 3 articles contained data that was not extractable, 8 articles did not include a comparison of the surgical process and 2 articles contained another type of pancreatectomy. Finally, 27 studies matched the inclusion criteria and were suitable for meta-analysis.^[7–10,21–43]^ A total of 6909 patients were included: 1306 patients in the MIPD group and 5603 patients in the OPD group. A PRISMA flow diagram depicting the selection process is shown in Figure 1. General study characteristics are detailed in Table 1. There were no RCTs identified. Expect for one descriptive study by Spheicher,^[25]^ all of the studies found were retrospective reviews that compared consecutive cases of minimally invasive PD with either consecutive or matched open procedures performed during the same time period in same center. The International Study Group of Pancreatic Surgery (ISGPS) definitions of postoperative pancreatic fistula (POPF) and DGE were used in the studies. A drain output of any measurable volume of fluid on or after postoperative day 3 with an amylase content >3 times the serum amylase activity was regarded as pancreatic fistula.^[44]^ DGE was defined as the need for maintenance of the nasogastric tube for 3 days or the inability to tolerate a solid diet by the seventh postoperative day.^[45]^ Two reviewers achieved perfect consensus in applying the eligibility criteria. For nonrandomized studies, the Newcastle-Ottawa quality assessment tool was employed to assess the study quality and the risk of publication bias. Newcastle-Ottawa Scale scores were >6 in 27 studies, indicating that all of the included articles were of high quality.^[46]^

**Table 1:**
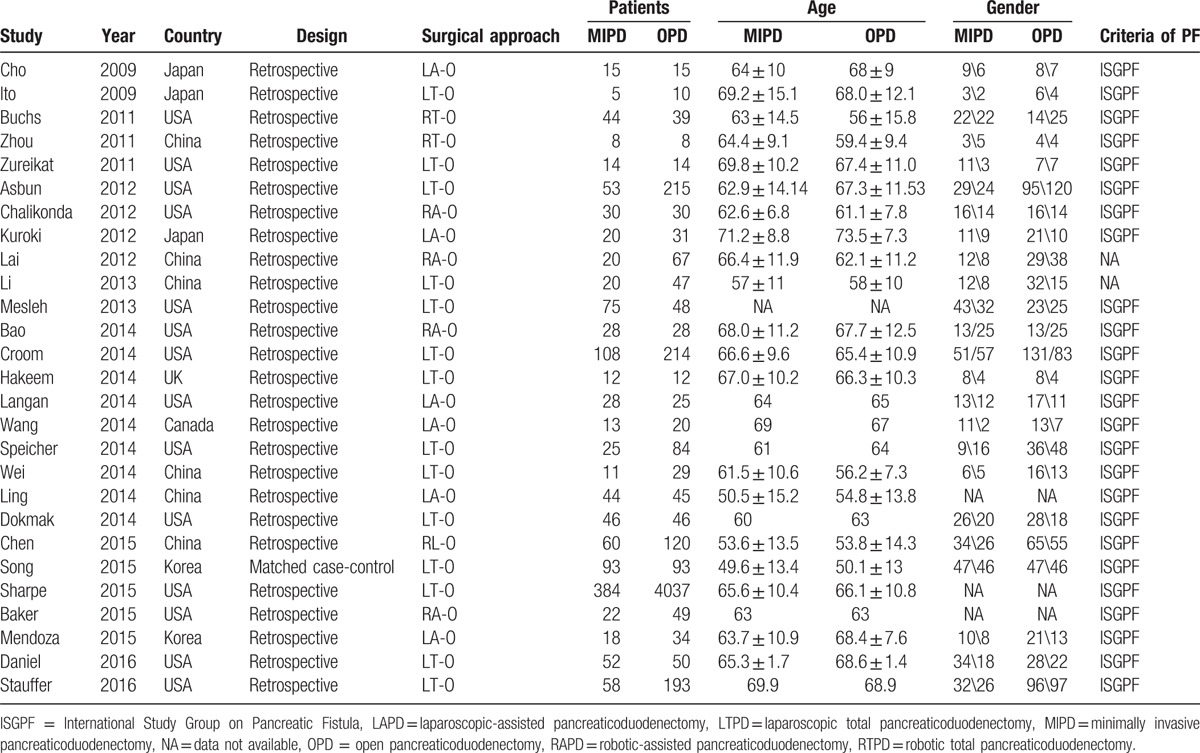
The general characteristic of the included studies.

### 3.2 Primary outcomes evaluation

The primary outcomes for this meta-analysis included POPF rate, wound infection, DGE rate, postoperative hemorrhage, mortality, and bile leakage.

### 3.3 Postoperative pancreatic fistula

**Figure 2:**
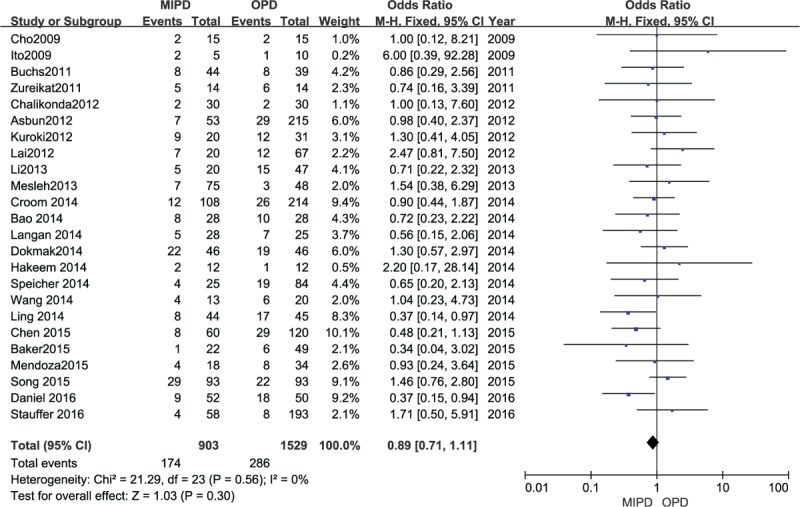
Forest plot and meta-analysis of pancreatic fistula, comparing MIPD with OPD.

All studies except those by Wei,^[27]^ Sharpe,^[30]^ and Zhou^[41]^ reported the incidence of POPF. The result of the meta-analysis including 2432 patients indicated that there was no significant difference in POPF rate between the MIPD and OPD groups (OR 0.89, 95% CI 0.71–1.11; *P* = .30, *I*^2^ = 0%) (Fig. 2).

### 3.4 Wound infection

**Figure 3:**
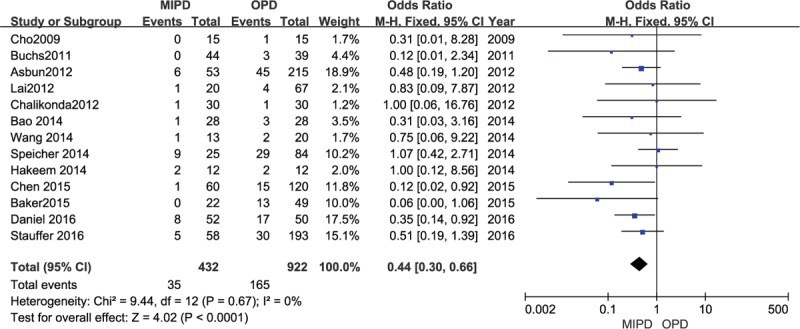
Forest plot and meta-analysis of wound infection, comparing MIPD with OPD.

Pooling data from 13 studies including 1354 patients proved that patients who underwent a minimally invasive approach suffered fewer wound infections compared with the OPD group (OR 0.44, 95% CI 0.30–0.66, *P* < .0001, *I*^2^ = 0%) (Fig. 3).

### 3.5 Delayed gastric emptying

Eighteen studies including 2027 patients reported the incidence of DGE in minimally invasive and open groups, which remained nonsignificantly different (OR, 0.76; 95% CI 0.56–1.02; *P* = .07, *I*^2^ = 0%).

### 3.6 Postoperative hemorrhage

**Figure 4:**
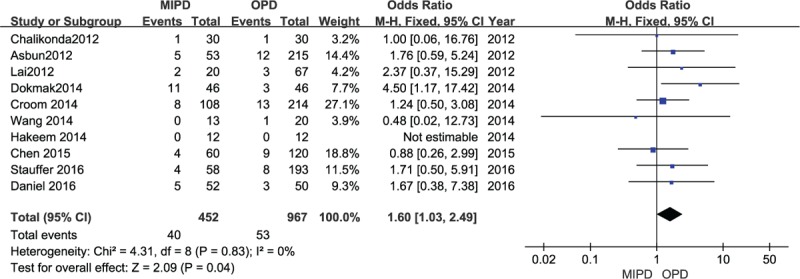
Forest plot and meta-analysis of postoperative hemorrhage, comparing MIPD with OPD.

Ten trials (1395 participants) provided data for the meta-analysis on this endpoint. The MIPD group showed a lower postoperative hemorrhage rate compared with the OPD group (OR 1.60, 95% CI 1.03–2.49; *P* = .04, *I*^2^ = 0%) (Fig. 4).

### 3.7 Mortality

Sixteen trials (5951 participants) provided data for the meta-analysis on this endpoint with no significant difference between the MIPD and OPD groups (OR 1.03, 95% CI 0.71–1.49; *P* = .88, *I*^2^ = 0%).

### 3.8 Bile leakage

Due to incomplete data, we extracted data about bile leakage in only 9 studies. The result of the meta-analysis indicated no evidence of a difference between the MIPD and OPD groups (OR 0.99, 95% CI 0.51–1.93; *P* = .98, *I*^2^ = 0%).

### 3.9 Secondary outcomes evaluation

The secondary outcomes included operative time, intraoperative estimated blood loss (EBL), the transfusion rate, the length of hospital stay (LOS), and the reoperation rate. As for oncological outcomes, lymph nodes harvested, R0 resection, tumor size, and vascular resection were evaluated.

### 3.10 Operative time

**Figure 5:**
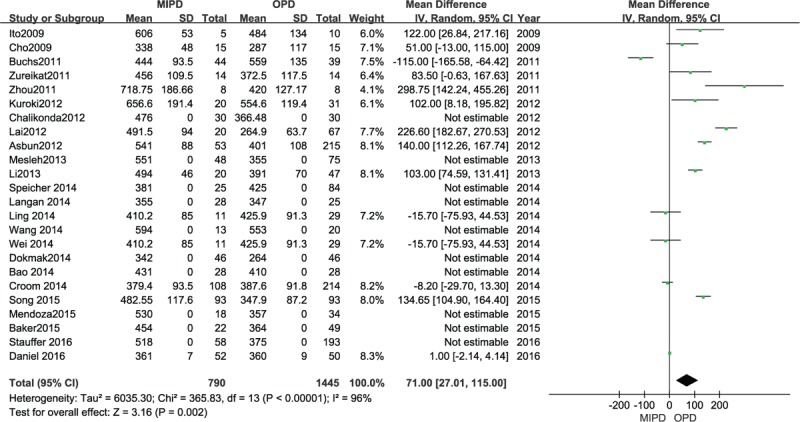
Forest plot and meta-analysis of operation time, comparing MIPD with OPD.

Twenty-four trials containing 2235 participants provided data for this analysis. When a random-effects model was used, operative time was significantly longer for MIPD (MD 71.00 minutes, 95% CI 27.01–115.00 minutes; *P* = .002, *I*^2^ = 96%) (Fig. 5).

### 3.11 Estimated blood loss

**Figure 6:**
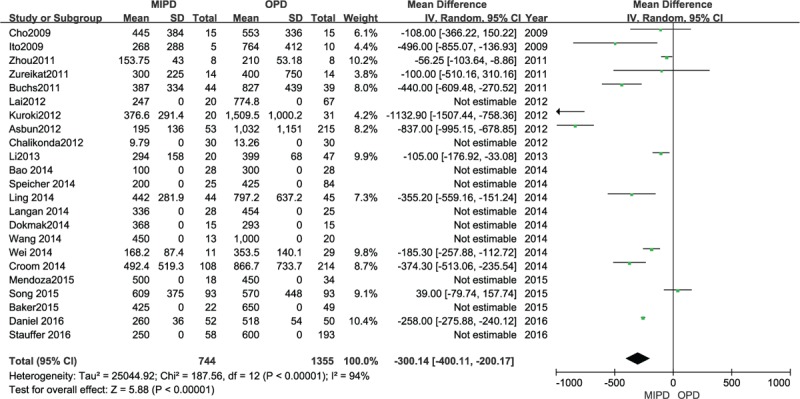
Forest plot and meta-analysis of EBL, comparing MIPD with OPD.

Twenty-three of the included studies reported the EBL for both procedures. The result of the meta-analysis indicated that minimally invasive surgery was associated with a reduction in intraoperative blood loss (MD −300.14 mL, 95% CI −400.11 to −200.17 mL; *P* < .00001, *I*^2^ = 94%) (Fig. 6).

### 3.12 Transfusion rate

**Figure 7:**
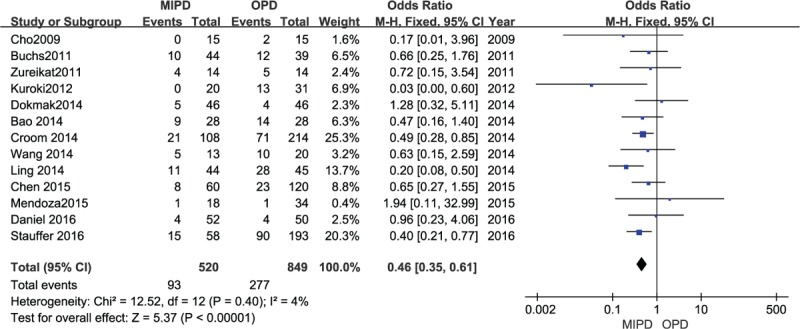
Forest plot and meta-analysis of transfusion rate, comparing MIPD with OPD.

Transfusion rate, available in 13 studies, manifested that the minimally invasive approach significantly minimized the transfusion rate (OR 0.46, 95% CI 0.35–0.61; *P* < .00001, *I*^2^ = 4%) (Fig. 7).

### 3.13 Length of hospital stay

**Figure 8:**
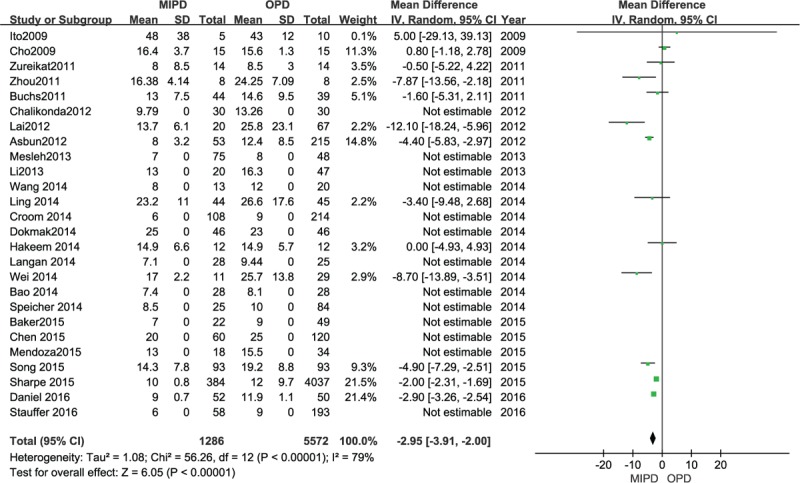
Forest plot and meta-analysis of length of hospital days.

The length of hospital stay was mentioned in all included studies except Kuroki.^[35]^ The meta-analysis showed that those undergoing MIPD demonstrated a shorter LOS than those undergoing OPD, and the difference was statistically significant (MD −2.95 days, 95% CI −3.91 to −2.00 days, *P* < .00001, *I*^2^ = 79%) (Fig. 8).

### 3.14 Lymph nodes harvested and R0 resection

**Figure 9:**
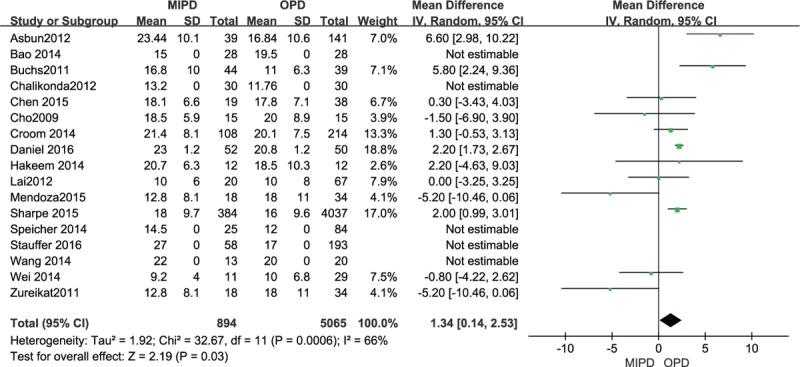
Forest plot and meta-analysis of lymph node harvested, comparing MIPD with OPD.

**Figure 10:**
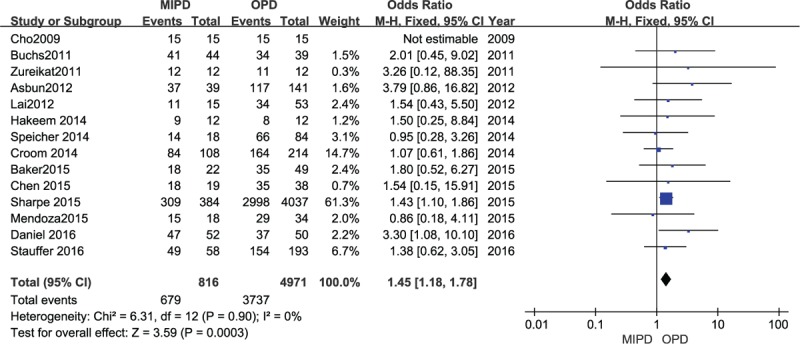
Forest plot and meta-analysis of R0 resection, comparing MIPD with OPD.

Ten included studies reported retrieved lymph nodes and the result of the meta-analysis showed that patients who underwent MIPD had more lymph nodes harvested than those who underwent OPD, and the difference was statistically significant (MD 1.34, 95% CI 0.14–2.53, *P* = .03, *I*^2^ = 66%) (Fig. 9). Fourteen studies including 5787 patients reported R0 resection in both groups, and the result of the meta-analysis demonstrated that the MIPD group had a higher R0 resection rate compared with the OPD group (OR 1.45, 95% CI 1.81–1.78, *P* = .0003, *I*^2^ = 0%) (Fig. 10).

### 3.15 Tumor size and vascular resection

The tumor size was mentioned in 12 studies and the meta-analysis showed there was no significant statistical difference between MIPD and OPD (MD −0.25, 95% CI −0.58 to 0.09, *P* = .15, *I*^2^ = 93%). For the vascular resection mentioned in only 5 studies, the meta-analysis also showed no significant difference (OR 0.93, 95% CI 0.65–1.33, *P* = .68, *I*^2^ = 0%).

### 3.16 Reoperation

Fourteen studies involving 6012 patients who mentioned reoperation rate showed no significant difference between MIPD and OPD groups (OR 0.73, 95% CI 0.50–1.08, *P* = .11, *I*^2^ = 0%).

### 3.17 Results of subgroup meta-analysis from high-volume centers

The literature included in our meta-analysis came from different centers with different sample sizes, which caused center bias. We selected the literature from high-volume centers, which included >50 patients in the OPD group. The primary and secondary outcomes were similar to the overall meta-analysis. The results of the subgroup meta-analysis indicated a similar conclusion. The results indicated that MIPD was associated with a reduction in wound infection (OR 0.45, 95% CI 0.25–0.84, *P* = .01), less EBL (MD −351.09, 95% CI −602.61 to −99.57, *P* = .006), a lower transfusion rate (OR 0.5, 95% CI 0.35–0.73, *P* = .0002), a shorter LOS (MD −2.47 days, 95% CI −2.70 to −2.24, *P* = .03), a higher R0 resection rate (MD 1.42, 95% CI 1.15–1.76, *P* = .001), and more lymph nodes harvested (MD 2.09, 95% CI 1.16–3.02, *P* < .00001). However, MIPD has a much longer operative time (MD 96.95 minutes, 95% CI 22.66–171.24, *P* = .01). Finally, there were no significant differences between the 2 procedures in POPF (*P* = .55), DGE (*P* = .02), postoperative hemorrhage (*P* = .17), bile leakage (*P* = .21), mortality (*P* = .16), tumor size (*P* = .20), vascular resection (*P* = .31), or reoperation rate (*P* = .27).

### 3.18 Assessment of methodologic quality and bias

There were no RCTs for inclusion in this study; therefore, the Cochrane Collaboration's tool for assessing risk of bias could not be applied. The studies included in our meta-analysis represent the experience of a single center or a single surgeon. They also correspond to the initial experience with a minimally invasive technique and do not consider the learning curve.

### 3.19 Sensitivity analysis and publication bias

**Figure 11:**
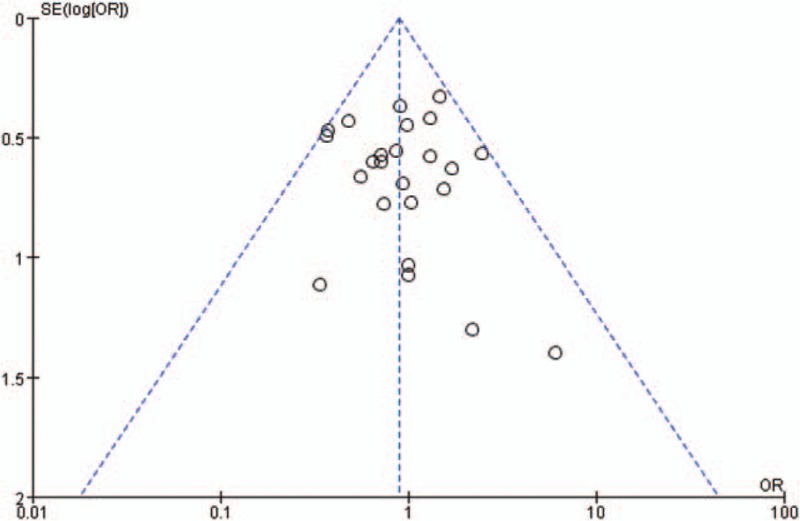
Funnel plot of complications in included studies, showing no publication bias.

Sensitivity analysis was conducted by eliminating each study included in the meta-analysis individually. However, there were no statistically significant changes about conclusions. Funnel plots were used to evaluate the publication bias, and the results indicated that there was no evident bias (Fig. 11).

## 4 Discussion

The development of minimally invasive techniques represents one of the most important advances in surgery during recent decades. Compared with open surgery, use of minimally invasive approach in colectomy, distal pancreatectomy, and hepatectomy procedures permitted reductions in blood loss and transfusion rates.^[47]^ Laparoscopic distal pancreatectomy has gained rapid acceptance, especially because of the low rate of intraoperative complications, short operative duration, and low morbidity.^[48]^ Efforts to extend the minimal access approach to more complex procedures have reached pancreaticoduodenectomy. With the development of computer science and technology, Giulianotti^[6]^ performed the first robotic Whipple procedure in 2003, and then the robotic surgical system was introduced into the field of pancreatic surgery. As a new technique, MIPD for pancreatic surgery is still controversial, and information regarding the comparison of using minimally invasive procedures over conventional OPD is rare. Therefore, a deliberate evaluation and meta-analysis using the latest data of published studies was made to compare the outcomes of MIPD and OPD. Although some centers have already conducted similar meta-analyses, our meta-analysis contains the latest and most comprehensive literature.

The results of this meta-analysis suggest advantages of MIPD over OPD in several aspects. First, MIPD was associated with a reduction in postoperative hemorrhage and wound infection. MIPD was also associated with less EBL, a lower transfusion rate, a shorter length of hospital stay, a higher R0 resection rate and more lymph nodes harvested. In contrast, the minimally invasive approach also had a much longer operative time. Finally, there were no significant differences between the 2 procedures in POPF, DGE, bile leakage, mortality, tumor size, vascular resection or reoperation rate. In the subgroup meta-analysis, the results were similar except that there was no difference in postoperative hemorrhage.

In this meta-analysis, the clear shortcoming of MIPD was the need of a long operation time, which could hinder the surgical efficiency. The operative time was significantly higher for MIPD than OPD, a common feature observed in other laparoscopic procedures, which mainly depends on the surgeon's experience and case load.^[49]^ There were several steps that affected the operation time, such as the complex dissection of pancreatic uncinate, the accurate anastomosis of alimentary canal and the hemorrhage control from major vasculature. Considering the factor of experience, surgeons who perform more MIPD may be more skillful, and the outcomes from Buchs^[9]^ are well explained, who reported that the operative time they spent on laparoscopic manners was lower than that for open procedure. Therefore, the proficiency of surgeons, development of surgical equipment, and preoperative estimation are significant factors to minimize the operative time.^[50,51]^

The most severe complications of pancreaticoduodenectomy are POPF, which have a close correlation with recovery. Our meta-analyses of 11 studies including 2432 patients showed there was no significant difference in POPF rate between the MIPD group and the OPD group. POPF is a major problem after pancreatic surgery which could cause other severe complications. However, the texture of the pancreas and the diameter of the main pancreatic duct were closely related to pancreatic fistula.^[44]^ In most cases of our analysis, authors neglect these 2 factors which could influence the quality of the result. In accordance with our study, the result of POPF in the former meta-analysis about same topic also showed no significant difference.^[50,52,53]^

Minimally invasive approach has been proved to be related with lower wound infection rates in abdominal surgery,^[54]^ such as cholecystectomy, colectomy, and gastrectomy. Our meta-analysis of 7 studies including 755 patients proved that patients who underwent surgery with a minimally invasive approach suffered fewer wound infections compared with the OPD group. As is known to all, the minimally invasive approach would limit the contamination of the surgical site because of the minor abdominal incision. Although other articles failed to draw the same conclusion, considering that information about definitions of wound infection as well as antibiotics used in the perioperative period were not mentioned in most studies, this question need to be investigated by RCT studies.

Postoperative hemorrhage is one of the most common complications in every surgical operation. In our meta-analysis, patients with MIPD suffered fewer postoperative hemorrhages compared with the OPD group. Perhaps patients who receive minimally invasive surgery may be selected by surgeons, such as patients who are not prone to bleeding or without vascular invasion received minimally invasive surgery. However, this finding also indicates that minimally invasive surgery can achieve the same effect on hemorrhage control as open techniques such as suture or ligation. In addition, our data of meta-analysis suggested there were no differences in other complications between 2 groups, such as DGE rate, mortality, and bile leakage. A well-designed randomized, prospective study comparing the open versus laparoscopic surgical approach would likely not be practicable because of the inadequate of standardization and intrinsic complexities of the pancreatic disease.^[40]^

Operative blood loss was shown in the meta-analysis to be lower in the MIPD group. It is true that the magnified view afforded by laparoscopy enhances the surgeon's view of the structures surrounding the specimen. With the help of laparoscopic instruments, surgeons could achieve precise dissection along appropriate planes, especially during dissection of the plane between the pancreatic uncinate and the superior mesenteric vessels. This explains the reason that OPD patients experienced much operative blood loss than MIPD patients. It also needs to be noted that during the preoperative assessment, those with expected bleeding were directly assigned to receive an open PD by the surgeons.^[55]^ Comparison of the operative blood loss required high-quality articles with matched cases, which could eliminate the selection bias to the greatest extent.

As for oncologic outcomes, R0 status and lymph node retrieval can be used as indicators of the postoperative prognosis.^[50]^ Our meta-analysis showed that MIPD had a significantly higher R0 resection rate and more lymph nodes were harvested in the MIPD group compared with the OPD group. The visual magnification provided by MIPD may allow for better lymph node clearance.^[52]^ Besides, our meta-analysis showed no significant differences in tumor size between the MIPD group and the OPD group. It is probable that larger tumors were operated using the open approach, and use of MIPD was prepared for patients with smaller lesions.

Another benefit of MIPD is a shorter hospital stay, which was proven by our data. Patients in the MIPD group had an average hospital stay that was 2.86 days shorter than the OPD group. As for cost-effective data, there were seldom included studies that described it in both groups. Mesleh reported that laparoscopic PD demonstrated an equivalent overall cost compared with OPD. Although operating time and supply costs were higher, this was balanced by the decreased cost of postoperative admission.^[37]^ There was no comparison in surgical prognosis between these 2 manipulations. Most of the studies focused on clinical treatment; only a small proportion obtained real-time follow-up. Therefore, we could not conduct the meta-analysis for short-term or long-term prognosis because of the lack of prognostic data from our original literature.

This meta-analysis has some limitations that must be taken into account. First, there were no RCTs, and no prospective studies of high quality that provided unbiased data for our analysis. This is, however, not only a limitation of our study, but an important indication that the widespread application of these techniques is yet to be achieved. Second, there was inevitably a selection bias in the published literature, as the baseline characteristics of patients and the indications for operative procedures in the 2 groups were not equal in all studies. It is also better to compare results of MIPD and OPD with risk stratification, but the available data was inadequate. In addition, patient preference, surgeon referral patterns, and evaluation of resectability could have introduced bias into selection of the approach. Sometimes, surgeons preferred to perform MIPD in healthy patients with fewer comorbidities, smaller lesions, a dilated common bile duct, and lower body mass index. Third, we applied a random-effects model to take between-study variation into consideration. Another major limitation lies in the possibility of publication bias, in which centers and individual surgeons who have had positive outcomes with MIPD are more likely to publish their findings. For each center, studies with significant results are more likely to get published than those with nonsignificant results. Some great pancreatic centers conduct a high volume of laparoscopic surgery and report their positive minimal approach findings. On the other hand, high-volume pancreatic centers regularly perform conventional open surgery and would report better results for OPD, and there is no doubt that their data would support OPD. Therefore, we performed a subgroup meta-analysis of high-volume centers and drew a similar conclusion, which aimed to reduce the influence of center bias. Finally, the cost comparison of MIPD versus OPD was not assessed in all studies. More evidence of prospective, multicenter, RCTs is needed to further address the true role of a minimally invasive technique in pancreatic surgery.

## 5 Conclusions

The findings of this meta-analysis suggest that MIPD was associated with fewer postoperative hemorrhages, fewer wound infections, longer operative time, less EBL, lower transfusion rate, shorter length of hospital stay, higher R0 resection rate, and more lymph nodes harvested compared with OPD. The minimally invasive approach can be a reasonable alternative to open PD with potential advantages. A selected population of patients treated with MIPD will show better surgical outcomes. RCTs or prospective cohort studies, which avoid selection and experimental bias and control for confounding factors, are necessary to adequately evaluate this question before routine application can be recommended.

## References

[1] Park KB Kwon OK Yu W Body composition changes after totally laparoscopic distal gastrectomy with delta-shaped anastomosis: a comparison with conventional Billroth I anastomosis. Surg Endosc 2016;30:4286–93. 26823058 10.1007/s00464-016-4744-x

[2] Huscher CG Mingoli A Sgarzini G Laparoscopic versus open subtotal gastrectomy for distal gastric cancer: five-year results of a randomized prospective trial. Ann Surg 2005;241:232–7. 15650632 10.1097/01.sla.0000151892.35922.f2 PMC1356907

[3] Seiler CA Wagner M Bachmann T Randomized clinical trial of pylorus-preserving duodenopancreatectomy versus classical Whipple resection-long term results. Br J Surg 2005;92:547–56. 15800958 10.1002/bjs.4881

[4] Whipple AO Parsons WB Mullins CR Treatment of carcinoma of the ampulla of Vater. Ann Surg 1935;102:763–79. 17856666 10.1097/00000658-193510000-00023 PMC1391173

[5] Gagner M Pomp A Laparoscopic pylorus-preserving pancreatoduodenectomy. Surg Endosc 1994;8:408–10. 7915434 10.1007/BF00642443

[6] Giulianotti PC Coratti A Angelini M Robotics in general surgery: personal experience in a large community hospital. Arch Surg 2003;138:777–84. 12860761 10.1001/archsurg.138.7.777

[7] Lai EC Yang GP Tang CN Robot-assisted laparoscopic pancreaticoduodenectomy versus open pancreaticoduodenectomy--a comparative study. Int J Surg 2012;10:475–9. 22732431 10.1016/j.ijsu.2012.06.003

[8] Chalikonda S Aguilar-Saavedra JR Walsh RM Laparoscopic robotic-assisted pancreaticoduodenectomy: a case-matched comparison with open resection. Surg Endosc 2012;26:2397–402. 22437947 10.1007/s00464-012-2207-6

[9] Buchs NC Addeo P Bianco FM Robotic versus open pancreaticoduodenectomy: a comparative study at a single institution. World J Surg 2011;35:2739–46. 21947494 10.1007/s00268-011-1276-3

[10] Cho A Yamamoto H Nagata M Comparison of laparoscopy-assisted and open pylorus-preserving pancreaticoduodenectomy for periampullary disease. Am J Surg 2009;198:445–9. 19342003 10.1016/j.amjsurg.2008.12.025

[11] Moher D Liberati A Tetzlaff J Preferred reporting items for systematic reviews and meta-analyses: the PRISMA statement. Int J Surg 2010;8:336–41. 20171303 10.1016/j.ijsu.2010.02.007

[12] Stroup DF Berlin JA Morton SC Meta-analysis of observational studies in epidemiology: a proposal for reporting. Meta-analysis Of Observational Studies in Epidemiology (MOOSE) group. JAMA 2000;283:2008–12. 10789670 10.1001/jama.283.15.2008

[13] Clarke M Horton R Bringing it all together: Lancet-Cochrane collaborate on systematic reviews. Lancet 2001;357:1728. 10.1016/S0140-6736(00)04934-5 11403806

[14] Hozo SP Djulbegovic B Hozo I Estimating the mean and variance from the median, range, and the size of a sample. BMC Med Res Methodol 2005;5:13. 15840177 10.1186/1471-2288-5-13 PMC1097734

[15] Lau J Ioannidis JP Schmid CH Quantitative synthesis in systematic reviews. Ann Intern Med 1997;127:820–6. 9382404 10.7326/0003-4819-127-9-199711010-00008

[16] Higgins JP Thompson SG Deeks JJ Measuring inconsistency in meta-analyses. BMJ 2003;327:557–60. 12958120 10.1136/bmj.327.7414.557 PMC192859

[17] Mantel N Mocarelli P Marocchi A Stratified analysis of multivariate clinical data: application of a Mantel-Haenszel approach. Stat Med 1983;2:259–66. 6648140 10.1002/sim.4780020221

[18] DerSimonian R Meta-analysis in the design and monitoring of clinical trials. Stat Med 1996;15:1237–48. 8817798 10.1002/(SICI)1097-0258(19960630)15:12<1237::AID-SIM301>3.0.CO;2-N

[19] Begg CB Mazumdar M Operating characteristics of a rank correlation test for publication bias. Biometrics 1994;50:1088–101. 7786990

[20] Egger M Davey SG Schneider M Bias in meta-analysis detected by a simple, graphical test. BMJ 1997;315:629–34. 9310563 10.1136/bmj.315.7109.629 PMC2127453

[21] Bao PQ Mazirka PO Watkins KT Retrospective comparison of robot-assisted minimally invasive versus open pancreaticoduodenectomy for periampullary neoplasms. J Gastrointest Surg 2014;18:682–9. 24234245 10.1007/s11605-013-2410-3

[22] Croome KP Farnell MB Que FG Total laparoscopic pancreaticoduodenectomy for pancreatic ductal adenocarcinoma: oncologic advantages over open approaches? Ann Surg 2014;260:633–8. 25203880 10.1097/SLA.0000000000000937

[23] Hakeem AR Verbeke CS Cairns A A matched-pair analysis of laparoscopic versus open pancreaticoduodenectomy: oncological outcomes using Leeds Pathology Protocol. Hepatobiliary Pancreat Dis Int 2014;13:435–41. 25100130 10.1016/s1499-3872(14)60048-5

[24] Langan RC Graham JA Chin AB Laparoscopic-assisted versus open pancreaticoduodenectomy: early favorable physical quality-of-life measures. Surgery 2014;156:379–84. 24680859 10.1016/j.surg.2014.03.018

[25] Speicher PJ Nussbaum DP White RR Defining the learning curve for team-based laparoscopic pancreaticoduodenectomy. Ann Surg Oncol 2014;21:4014–9. 24923222 10.1245/s10434-014-3839-7

[26] Wang Y Bergman S Piedimonte S Bridging the gap between open and minimally invasive pancreaticoduodenectomy: the hybrid approach. Can J Surg 2014;57:263–70. 25078932 10.1503/cjs.026713 PMC4119119

[27] Wei H Wei B Zheng Z Comparative study of outcomes after laparoscopic versus open pancreaticoduodenectomy. Zhonghua Wei Chang Wai Ke Za Zhi 2014;17:465–8. 24859956

[28] Yun L QZCP Analysis of the relevant factors of pancreatic fistula after robot assisted pancreatic surgery. J Hepatobiliary Surg 2014;1:15–9.

[29] Chen S Chen JZ Zhan Q Robot-assisted laparoscopic versus open pancreaticoduodenectomy: a prospective, matched, mid-term follow-up study. Surg Endosc 2015;29:3698–711. 25761559 10.1007/s00464-015-4140-y

[30] Sharpe SM Talamonti MS Wang CE Early national experience with laparoscopic pancreaticoduodenectomy for ductal adenocarcinoma: a comparison of laparoscopic pancreaticoduodenectomy and open pancreaticoduodenectomy from the National Cancer Data Base. J Am Coll Surg 2015;221:175–84. 26095569 10.1016/j.jamcollsurg.2015.04.021

[31] Song KB Kim SC Hwang DW Matched case-control analysis comparing laparoscopic and open pylorus-preserving pancreaticoduodenectomy in patients with periampullary tumors. Ann Surg 2015;262:146–55. 25563866 10.1097/SLA.0000000000001079

[32] Stauffer JA Coppola A Villacreses D Laparoscopic versus open pancreaticoduodenectomy for pancreatic adenocarcinoma: long-term results at a single institution. Surg Endosc 2017;31:2233–41. 27604369 10.1007/s00464-016-5222-1

[33] Delitto D Luckhurst CM Black BS Oncologic and perioperative outcomes following selective application of laparoscopic pancreaticoduodenectomy for periampullary malignancies. J Gastrointest Surg 2016;20:1343–9. 27142633 10.1007/s11605-016-3136-9 PMC6033586

[34] Li YB Wang X Wang MJ Delayed gastric emptying after laparoscopic versus open pancreaticoduodenectomy: a comparative study. Zhonghua Wai Ke Za Zhi 2013;51:304–7. 23895749

[35] Kuroki T Adachi T Okamoto T A non-randomized comparative study of laparoscopy-assisted pancreaticoduodenectomy and open pancreaticoduodenectomy. Hepatogastroenterology 2012;59:570–3. 21940382 10.5754/hge11351

[36] Zureikat AH Breaux JA Steel JL Can laparoscopic pancreaticoduodenectomy be safely implemented? J Gastointest Surg 2011;15:1151–7. 10.1007/s11605-011-1530-x 21538192

[37] Mesleh MG Stauffer JA Bowers SP Cost analysis of open and laparoscopic pancreaticoduodenectomy: a single institution comparison. Surg Endosc 2013;27:4518–23. 23943116 10.1007/s00464-013-3101-6

[38] Ito M Horiguchi A Ishihara S Laparoscopic pancreatic surgery: totally laparoscopic pancreatoduodenectomy and reconstruction. Pancreas 2009;38:1009.

[39] Dokmak S Fteriche FS Aussilhou B Laparoscopic pancreaticoduodenectomy should not be routine for resection of periampullary tumors. J Am Coll Surg 2015;220:831–8. 25840531 10.1016/j.jamcollsurg.2014.12.052

[40] Asbun HJ Stauffer JA Laparoscopic vs open pancreaticoduodenectomy: overall outcomes and severity of complications using the Accordion Severity Grading System. J Am Coll Surg 2012;215:810–9. 22999327 10.1016/j.jamcollsurg.2012.08.006

[41] Zhou NX Chen JZ Liu QD Outcomes of pancreatoduodenectomy with robotic surgery versus open surgery. Int J Med Robot 2011;7:131–7. 21412963 10.1002/rcs.380

[42] Baker EH Ross SW Seshadri R Robotic pancreaticoduodenectomy: comparison of complications and cost to the open approach. Int J Med Robot 2016;12:554–60. 26202591 10.1002/rcs.1688

[43] Mendoza ASR Han HS Yoon YS Laparoscopy-assisted pancreaticoduodenectomy as minimally invasive surgery for periampullary tumors: a comparison of short-term clinical outcomes of laparoscopy-assisted pancreaticoduodenectomy and open pancreaticoduodenectomy. J Hepatobiliary Pancreat Sci 2015;22:819–24. 26455716 10.1002/jhbp.289

[44] Bassi C Dervenis C Butturini G Postoperative pancreatic fistula: an international study group (ISGPF) definition. Surgery 2005;138:8–13. 16003309 10.1016/j.surg.2005.05.001

[45] Wente MN Bassi C Dervenis C Delayed gastric emptying (DGE) after pancreatic surgery: a suggested definition by the International Study Group of Pancreatic Surgery (ISGPS). Surgery 2007;142:761–8. 17981197 10.1016/j.surg.2007.05.005

[46] Stang A Critical evaluation of the Newcastle-Ottawa scale for the assessment of the quality of nonrandomized studies in meta-analyses. Eur J Epidemiol 2010;25:603–5. 20652370 10.1007/s10654-010-9491-z

[47] Di Giuseppe P Ajmar R Carpal tunnel release using minimally invasive technique. Plast Reconstr Surg 1996;97:1310–1. 8628826 10.1097/00006534-199605000-00046

[48] Briggs CD Mann CD Irving GR Systematic review of minimally invasive pancreatic resection. J Gastrointest Surg 2009;13:1129–37. 19130151 10.1007/s11605-008-0797-z

[49] Kooby DA Chu CK Laparoscopic management of pancreatic malignancies. Surg Clin North Am 2010;90:427–46. 20362796 10.1016/j.suc.2009.12.011

[50] Lei P Wei B Guo W Minimally invasive surgical approach compared with open pancreaticoduodenectomy: a systematic review and meta-analysis on the feasibility and safety. Surg Laparosc Endosc Percutan Tech 2014;24:296–305. 24743678 10.1097/SLE.0000000000000054

[51] Kauffmann EF Napoli N Signori S Analysis of instrument traffic during laparoscopic robot-assisted pancreaticoduodenectomy. HPB 2012;13:689.

[52] Nigri G Petrucciani N La Torre M Duodenopancreatectomy: open or minimally invasive approach? Surgeon 2014;12:227–34. 24525404 10.1016/j.surge.2014.01.006

[53] Zhang H Wu X Zhu F Systematic review and meta-analysis of minimally invasive versus open approach for pancreaticoduodenectomy. Surg Endosc 2016;30:5173–84. 27005287 10.1007/s00464-016-4864-3

[54] Varela JE Wilson SE Nguyen NT Laparoscopic surgery significantly reduces surgical-site infections compared with open surgery. Surg Endosc 2010;24:270–6. 19533235 10.1007/s00464-009-0569-1

[55] Correa-Gallego C Dinkelspiel HE Sulimanoff I Minimally-invasive vs open pancreaticoduodenectomy: systematic review and meta-analysis. J Am Coll Surg 2014;218:129–39. 24275074 10.1016/j.jamcollsurg.2013.09.005

